# Endoscopic surgery versus craniotomy in the treatment of spontaneous intracerebral hematoma: a systematic review and meta-analysis

**DOI:** 10.1186/s41016-022-00304-1

**Published:** 2022-11-25

**Authors:** Xiaolin Du, Xiaoning Lin, Cheng Wang, Kun Zhou, Yigong Wei, Xinhua Tian

**Affiliations:** 1 Department of Neurosurgery, The Jinyang Hospital Affiliated to Guizhou Medical University, Guiyang, 550084 China; 2 Department of Neurosurgery, Zhong Shan hospital Xiamen University, Xiamen, 361012 China

**Keywords:** Intracerebral hematoma, Neuroendoscopy, Craniotomy

## Abstract

**Background:**

Spontaneous intracerebral hemorrhage (SICH) has high morbidity and mortality, with no clear standard of treatment available. Compared with the craniotomy approach, neuroendoscopy is a relatively minimally invasive treatment method, and may be an efficient alternative. Therefore, this meta-analysis aimed to assess the clinical efficacy of neuroendoscopy and craniotomy in SICH patients.

**Methods:**

The electronic databases Web of Science, PubMed, EmBase, MEDLINE, and the Cochrane Library were systematically searched. According to the PRISMA template, we finally selected and analyzed 14 eligible studies that evaluated neuroendoscopy versus craniotomy. Primary outcomes included operation time, intraoperative blood loss volume, evacuation rate, residual hematoma, complications, hospital stay duration, clinical outcomes, and other parameters.

**Results:**

A total of 4 randomized controlled trials (RCTs) and 10 retrospective studies (non-RCTs) involving 1652 patients were included in the final analysis. In the neuroendoscopy (NE) group, operation time (*p* < 0.00001), intraoperative blood loss volume (*p* < 0.0001), hematoma evacuation rate (*p* = 0.0002), complications (*p* < 0.00001), hospitalization days (*p* = 0.004), and mortality (*p* < 0.0001) were significantly different from those of the craniotomy (C) group, with a higher rate of good recovery compared with the craniotomy group (*P* < 0.00001).

**Conclusions:**

These findings suggest that patients with SICH and physicians may benefit more from neuroendoscopic surgery than craniotomy.

## Background

Intracerebral hemorrhage (ICH) accounts for 10–15% of all strokes in the USA, Europe and Australia, and 20–30% of Asian cases, with a 30-day mortality rate of 35% to 52%; half of the related deaths occur in the first 2 days [1–4]. Its overall incidence is 24.6 per 100,000 person-years, indicating that it represents the most fatal type of stroke around the world [5]. It is worth noting that most of individuals living with ICH have varying degrees of long-term disability. Only 20% of ICH patients survive independently within 6 months [3]. Its main risk factors include age, a history of hypertension, East and Southeast Asian ethnicity, smoking, drug, and alcohol abuse, inherited or acquired coagulopathies, anticoagulant use, past stroke history, vascular abnormalities (arteriovenous malformations, developmental venous abnormalities, amyloid angiopathy) and potential tumors [6]. Gender may also be a risk factor, although not statistically significant, but ICH incidence in women is 15% lower than in men [7]. Such high mortality and disability rates undoubtedly impose great mental and economic burden upon patients and their families.

Currently, ICH treatment options mainly include endoscopic evacuation, stereotactic aspiration, conventional craniotomy, and conservative treatment. Indeed, treatment of patients with ICH encounters two major problems. The first problem is treatment selection, i.e., conservative or surgical treatment; the second is the selection of the operation, i.e., endoscopic surgery or craniotomy. In general, patients with small hematomas and no neurological deficits tend to opt for conservative treatment, while surgery tends to be performed in those with massive hemorrhage and progressive neurological deterioration [8, 9]. In recent years, neuroendoscopic surgery for ICH has attracted much attention, because it is more minimally invasive than craniotomy, and can reduce the characteristic peripheral brain injury. An increasing number of physicians now select endoscopy for the treatment of ICH patients, but its long-term efficacy and complications remain controversial. Therefore, clarifying which surgical method between neuroendoscopy and craniotomy is more efficient for ICH patients is critical.

## Methods

### Search strategy

A literature search was performed by two independent investigators (Du and Wang) in various electronic databases, including PubMed, EmBase, MEDLINE, Cochrane Controlled Trials Register and Web of Science, from inception to July 2018, with the following keywords: “(Endoscope OR Endoscopy OR endoscopic surgery OR neuroendoscopic surgery) AND (Intracranial Hemorrhage OR Intracranial Hemorrhage, Hypertensive OR cerebral Hemorrhage OR brain Hemorrhage OR putaminal Hemorrhage OR basal ganglia Hemorrhage OR thalamic Hemorrhage OR subcortex Hemorrhage)”. After sequentially reviewing the titles, abstracts, and full texts of the retrieved reports according to the PRISM statement, studies clearly irrelevant were excluded. Any disagreement between the two investigators was resolved by consensus or a third investigator if required. The study authors were contacted for clarifications and further information when necessary. The search was limited to studies published in English.

### Inclusion and exclusion criteria

Inclusion criteria were (1) diagnosis of intracranial hemorrhage by computed tomography; (2) treatment methods included endoscopic surgery and craniotomy, with or without intralesional thrombolysis; (3) randomized controlled trials (RCTs) or prospective controlled studies (non-RCTs). Studies were excluded if they included or were (1) infratentorial intracerebral hemorrhage; (2) brain injury, bleeding due to brain tumor, and bleeding tendencies caused by uremia, liver cirrhosis, or anticoagulation therapy, intracranial aneurysm, cerebral arteriovenous malformation, subdural hemorrhage, extradural hemorrhage subarachnoid hemorrhage or pituitary apoplexy; (3) incomplete data or non-English publication; (4) meta-analyses, editorials, letters, errata, case reports, reviews, and animal experiments.

### Data extraction

The data were extracted by two investigators independently according to eligibility criteria, and included basic information (author name, year, and type of documents) and basic patient characteristics (gender and age, number of cases, hematoma location, hematoma evacuation rate, hematoma residual volume, intraoperative blood loss, operation time, hospitalization duration, postoperative complications, mortality, and good recovery). All data were recorded using an Excel sheet. Good functional outcome (GFO) was defined as a patient being able to care for him/herself, corresponding to a modified Rankin Scale (mRS) score of 0, 1, 2, or 3, a Glasgow Outcome Scale (GOS) score of 4 or 5, or activities of daily living (ADL) score of 1, 2, or 3, or a Barthel index (BI) > 60. If more than 1 scale was used to evaluate the patients’ functional outcomes within an article, we first selected the GOS as the assessment scale, and then the modified Rankin Scale, the BI, and ADL score. Any discrepancies were solved by discussion and consensus.

### Statistical analysis

Statistical analyses were performed with the Review Manager 5.3 software and forest plots were generated, with statistical significance defined as *p* < 0.05. Between studies heterogeneity was assessed by the standard chi-square test and *I*^2^ statistic; heterogeneity was pre-specified at *p* ≤ 0.10 or *I*^2^ ≥ 50% in this study. In case of low-moderate heterogeneity, a fixed-effects model was used for data analysis. Otherwise, a random-effects model was used to analyze the pooled data. Dichotomous variables were expressed as relative risk ratio (RR) with a 95% confidence interval (CI). Continuous variables were assessed using standard mean difference (SMD). All tests were two-tailed, and publication bias was assessed using funnel plots.

## Results

### Study selection

**Fig. 1:**
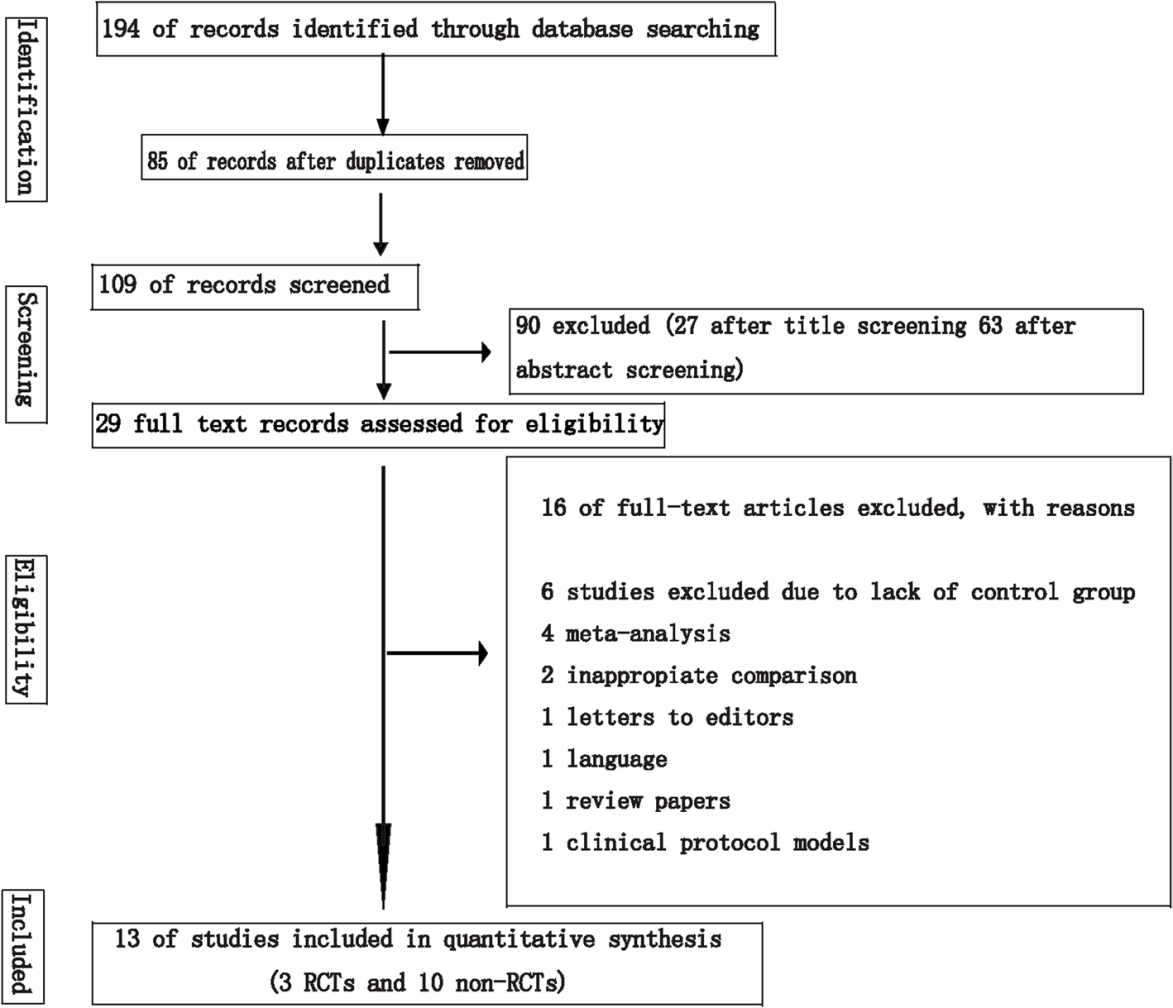
PRISMA flow diagram

The electronic search yielded 2526 hits in all databases; of which, 1419 were included after duplicates were removed. Title and abstract review were performed for the remaining 1419 articles, and 1257 hits were excluded. The full text of the remaining 62 articles were retrieved, and 48 were excluded for the following reasons: (1) lack of control group (*n* = 27); (2) meta-analysis (*n* = 8); (3) review papers (*n* = 4); (4) bleeding located in the cerebellum (3); (5) Clinical protocol models (*n* = 2); (6) full texts not available (*n* = 2);( 7) erratum (*n* = 1); (8) letter to editors (*n* = 1). Finally, 14 studies were included in the final analysis, comprising 4 RCTs [10–13] and 10 non-RCTs [14–23]) (Fig. 1).

### Main characteristics

The main characteristics of studies included study basic information and patient basic characteristics (Tables 1 and 2). Each trial described the baseline characteristics of the enrolled participants, with no significant differences between the neuroendoscopy(NE) and craniotomy(C) groups.

**Table 1:** Main characteristics

Study (year)	Study type	Number of patients	Age	ICH volume (mL)	ICH location
		(NE/C)	(NE/C)	(NE/C)	
Nakano (2003) [14]	non-RCT	6/11	55/62	< 40 ml VS 40 ml(64%)	Supratentorial
Qiu (2003) [15]	non-RCT	25/22	62/60	All > 30 ml	Supratentorial
Cho (2006) [10]	RCT	30/30	57/54	55.5 ± 23.3 VS 42.1 ± 18.4	Supratentorial
Zhu (2011) [16]	non-RCT	28/30	61/65	53.7 ± 15.8 VS 63.9 ± 17.0	Supratentorial
Chi (2014) [17]	non-RCT	144/610	63/63	58.2 ± 17.5VS 83.4 ± 27.5	Supratentorial
Zhang H (2014) [11]	RCT	21/30	60/61	58.3 ± 18.8 VS 62.2 ± 15.6	Supratentorial
Wang W (2015) [18]	non-RCT	21/24	57/57	61.2 VS 47.1	Supratentorial
Yamashiro (2015) [19]	non-RCT	14/8	70/58	131.7 ± 52.2 VS 99.2 ± 16.5	Supratentorial
Feng (2016) [12]	RCT	93/91	66/69	NA	Supratentorial
Cai (2017) [20]	non-RCT	20/21	60/57	51.7 ± 19.6 VS 56.3 ± 23.5	Supratentorial
Li Y (2017) [21]	non-RCT	32/31	61/62	54.5 ± 14.2 VS 59.9 ± 14.6	Supratentorial
Xu (2017) [22]	non-RCT	82/69	53/54	55.2 ± 28.4 VS 55.9 ± 27.6	Supratentorial
Eroglu (2018) [23]	non-RCT	17/17	56/54	53.1 ± 4.6VS51.5 ± 4.1	Supratentorial
Zhang J (2018) [13]	RCT	65/65	Mean = 62	39.1 ± 6.2 VS 39.0 ± 6.1	Supratentorial

**Table 2:** Main characteristics and quality assessment of the selected articles

Study (year)	Good recovery	Death	Follow-up	Outcome measurement	Quality score
	(NE/C)	(NE/C)			
Nakano (2003) [14]	4/2	0/0	NA	GOS	5
Qiu (2003) [15]	18/13	2/NA	6	GOS	7
Cho (2006) [10]	NA	0/4	6	BI	6
Zhu (2011) [16]	7/3	2/5	3	GOS	7
Chi (2014) [17]	111/319	15/14	3-6	ADL	7
Zhang H (2014) [11]	11/4	0/3	6-20	GOS	5
Wang W (2015) [18]	NA	2/1	6-12	GOS	7
Yamashiro (2015) [19]	5/4	0/1	NA	mRS	6
Feng (2016) [12]	60/39	6/8	6	ADL	6
Cai (2017) [20]	NA	1/3	Discharge	GCS	7
Li Y (2017) [21]	NA	6/8	6	GOS	7
Xu (2017) [22]	30/11	15/18	6	mRS	7
Eroglu (2018) [23]	NA	2/4	6	GOS	7
Zhang J (2018) [13]	59/39	0/0	3	GOS	7

*NE* endoscopic surgery, *C* Craniotomy, *RCT* Randomized controlled trial, *NA* Not available, *GOS* Glasgow Outcome Scale, *BI* Barthel Index, *ADL* activities of daily living score, *mRS* modified Rankin Scale, *GCS* Glasgow Coma Scale

The Cochrane criteria and Newcastle-Ottawa scale were used to assess the quality of RCT and non-RCT, respectively

Fourteen studies (4 RCTs and 10 non-RCTs) with a total of 1652 patients (598 and 1054 patients in the endoscopy and craniotomy groups, respectively) were included in the current meta-analysis of ICH treatment，among whom 9(4 and 5 patients in the endoscopy and craniotomy groups, respectively) were lost to follow-up [23]. Therefore, 1643 patients were finally being analyzed. The rate of patients lost to follow-up was within the permissible range. It should be noted that the endoscopic group included patients who underwent endoscopic surgery alone or in combination with stereotactic aspiration, e.g., Cho’s study [10]. The craniotomy group included large and small bone flap craniotomies. Large and small bone flap craniotomies in Chi^’^s article [17] were compared respectively, and were grouped into the craniotomy group. Three articles were excluded, including one [24] for subtentorial hematoma assessment; in the other two articles [25, 26], some of the patients were cerebellar hemorrhage cases, which would not significantly affect the outcomes of good recovery and mortality (data not shown).

### Quality assessment of the selected articles

**Fig. 2:**
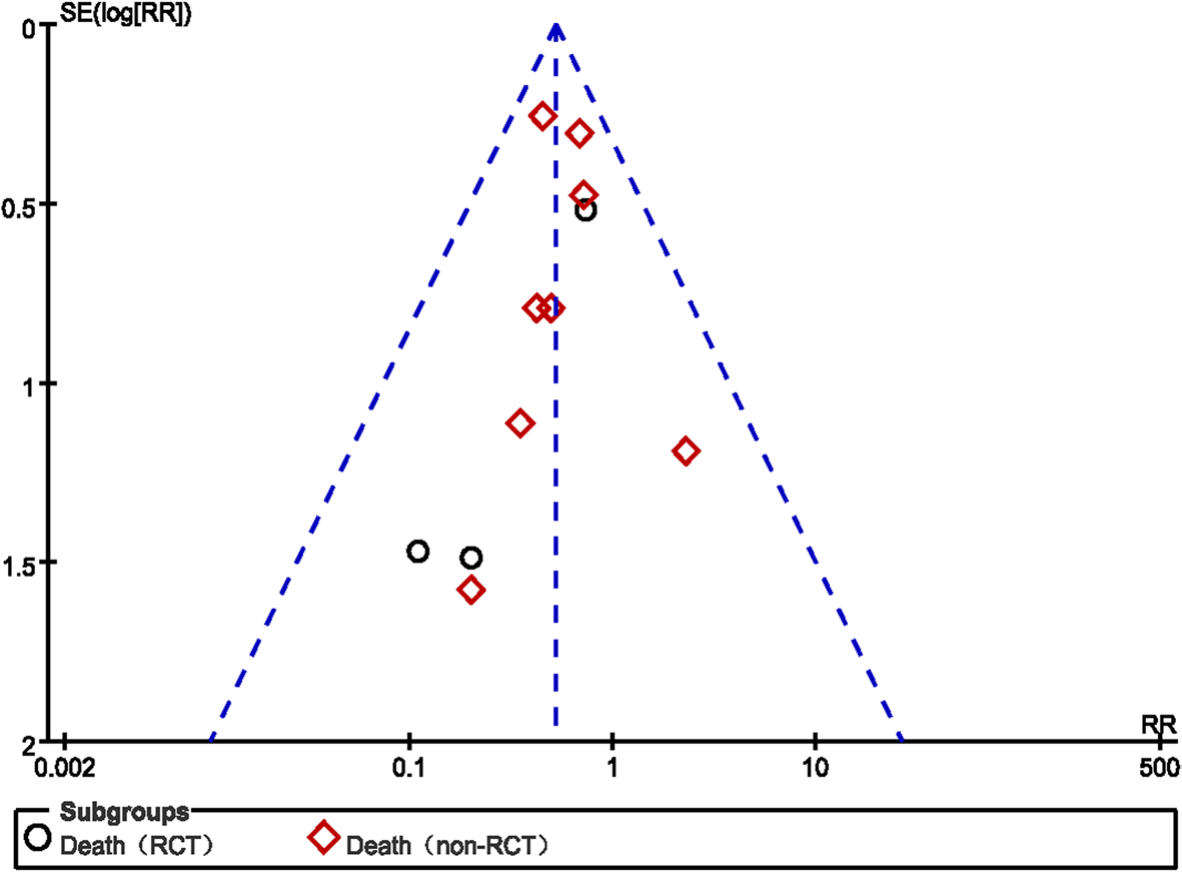
Mortality in NE and craniotomy groups. The funnel plot was visually symmetric

**Fig. 3:**
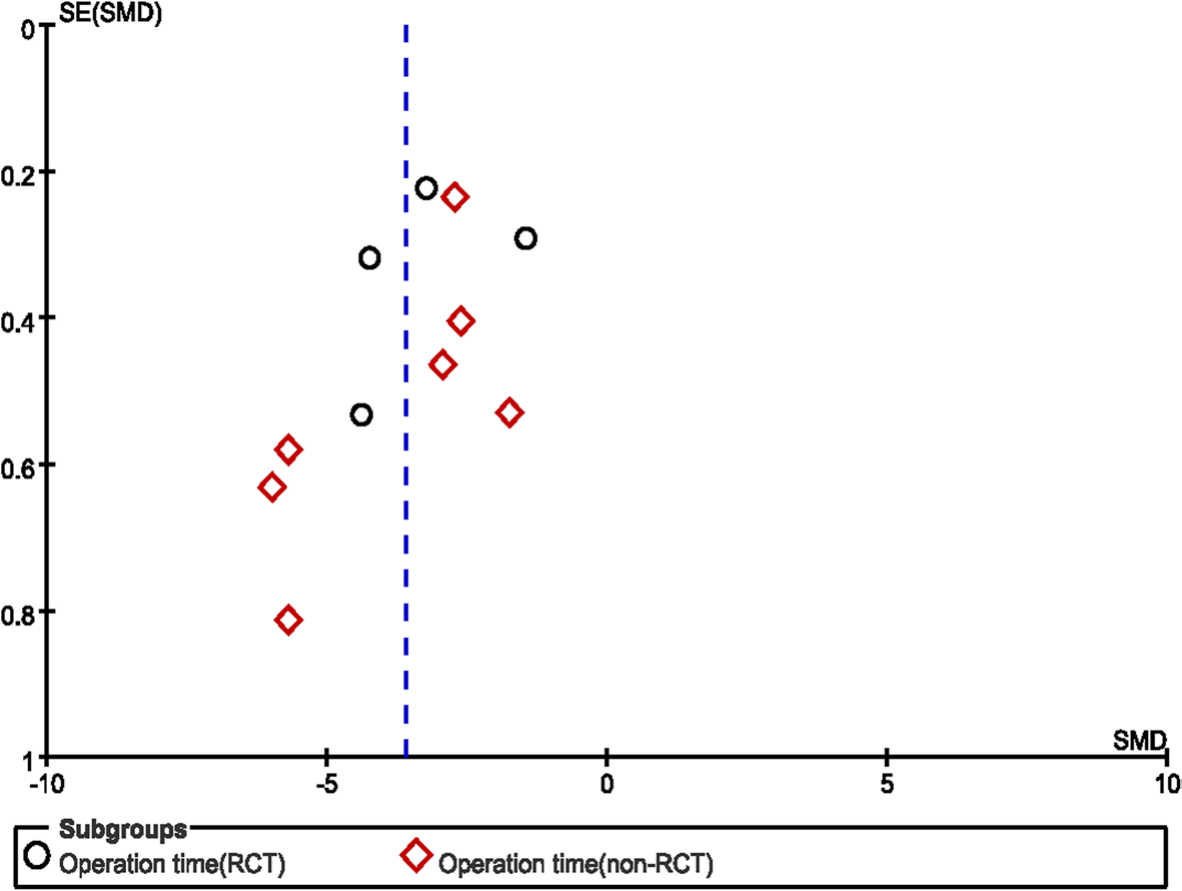
Duration of operation in the NE and craniotomy groups. The funnel plot was visually symmetric

The Cochrane criteria were used to assess the quality of RCTs: low risk indicated low risk bias of bias; high risk reflected high risk of bias; unclear risk indicated that the report did not provide sufficient or uncertain information for bias assessment. The Newcastle-Ottawa scale was used to assess the quality of non-TRCTs: a total score of < 5 reflected low-quality. Table 2 summarizes the risk of bias of all included studies. In addition, to test whether publication bias was present among trails included in this meta-analysis, we used funnel plots (Figs. 2 and 3). Although the total number of studies in this meta-analysis is small, distribution in the funnel plots is symmetrical. This result suggests that there is no publication bias.

### Pooled results

#### Rebleeding in the NE and C groups

**Fig. 4:**
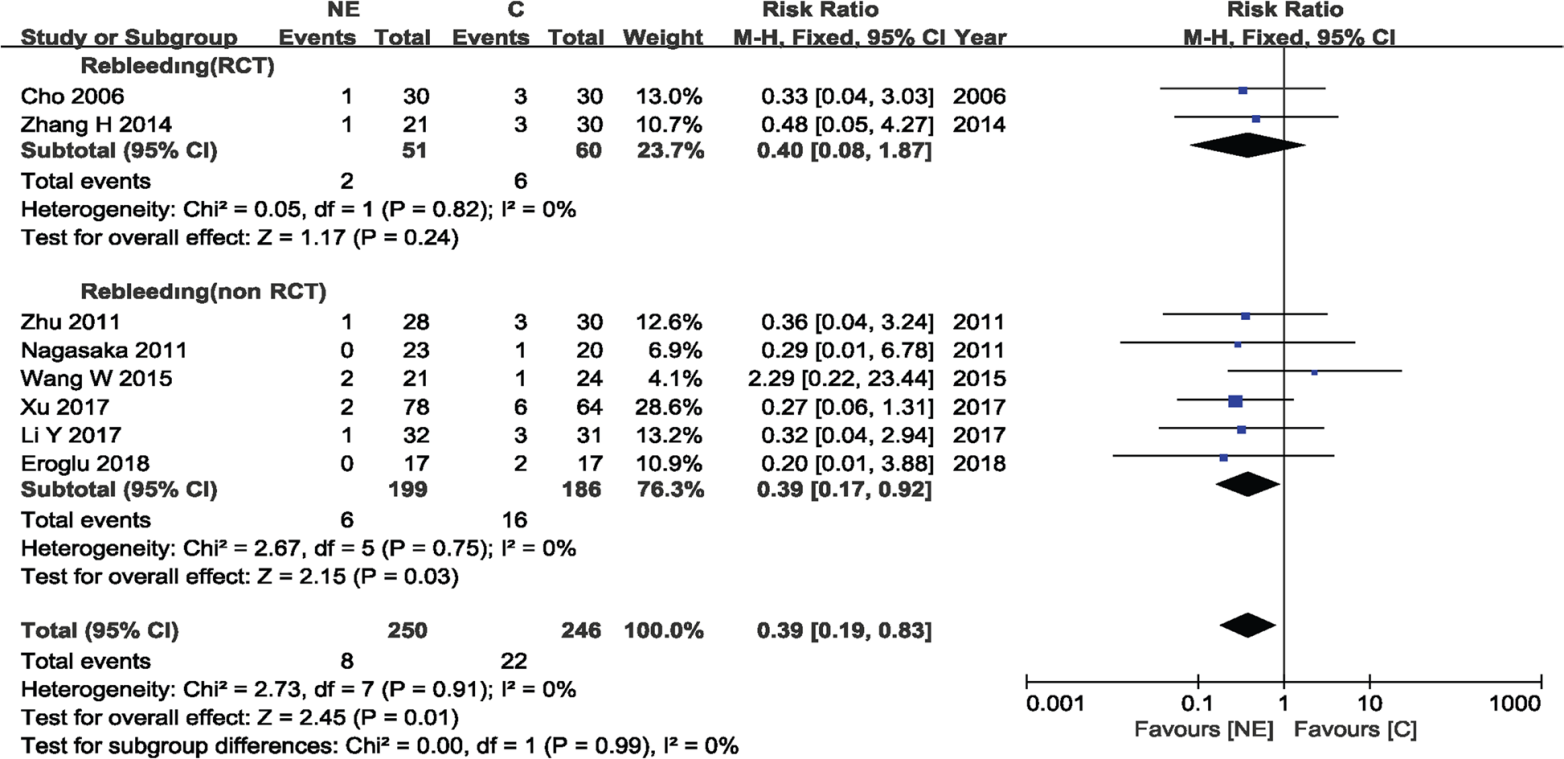
Rebleeding in the NE and C groups

In seven studies [10, 11, 16, 18, 21–23] including 453 participants, the rate of rebleeding was statistically significant in the non-RCT group and the overall effect, with RR = 0.40 (95%CI 0.17–0.98; *p* = 0.04) and RR = 0.40 (95%CI 0.19–0.87; *p* = 0.02), respectively; meanwhile, there was no significant difference in the RCT group, with RR = 0.40 (95%CI 0.08–1.87; *p* = 0.24). There was no evidence of statistically significant heterogeneity (RCTs, *p* = 0.82 and *I*^2^ = 0%; non-RCTs, *p* = 0.62 and *I*^2^ = 0%; overall, *p* = 0.85 and *I*^2^ = 0%), and a fixed-effects model was used for analysis. This finding was discordant with a previous publication [27]. In a forest plot, one article was excluded [20] because there was no case of rebleeding in the endoscopy and craniotomy groups (Fig. 4).

#### Evacuation rates in the NE and craniotomy groups

**Fig. 5:**
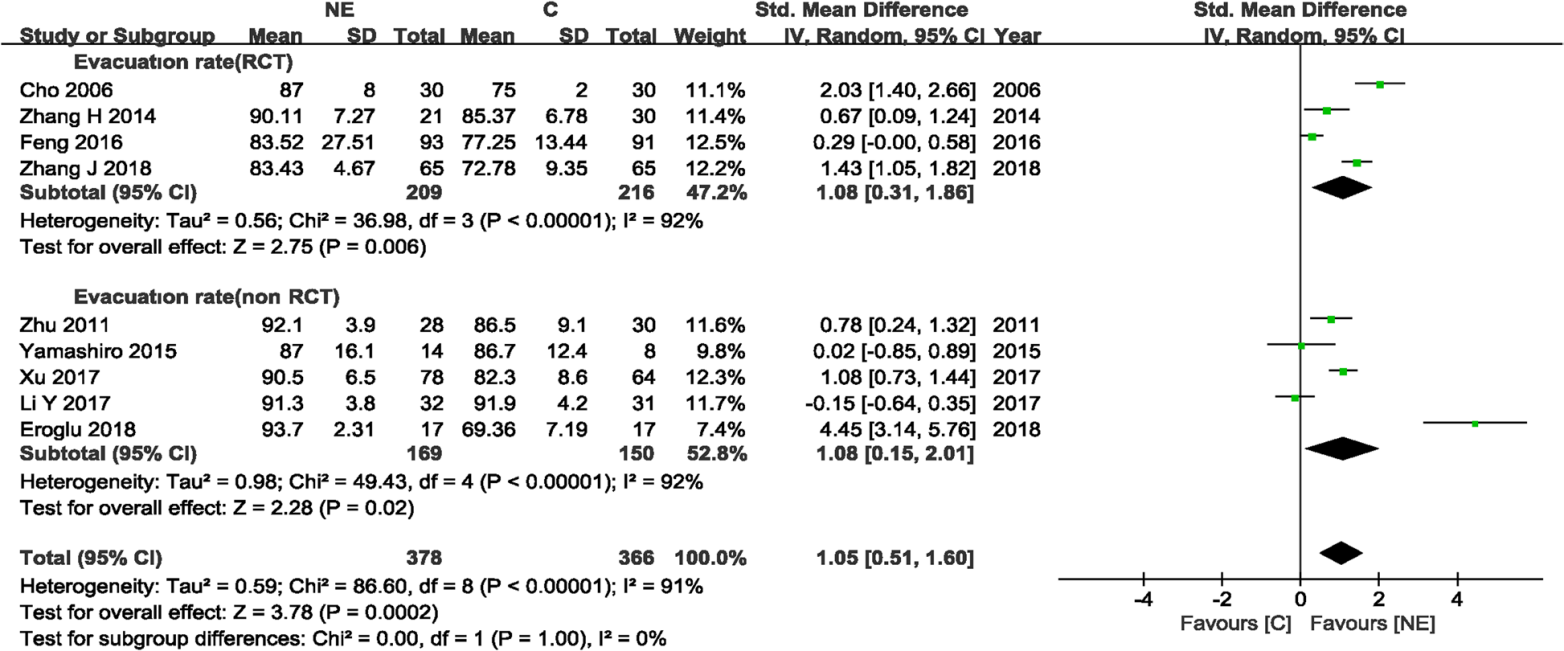
Evacuation rates in the NE and craniotomy groups

Nine trials [10–13, 16, 19, 21–23] (4 RCTs and 5 non-RCTs) evaluating 744 patients (378 and 366 patients in the experimental and control arms, respectively) assessed evacuation rates. There were significant differences in evacuation rate for the RCT and non-RCTs groups, with SMD = 1.08 (95%CI 0.31–1.86, *P* = 0.006) and SMD = 1.08 (95%CI 0.15–2.01, *P* = 0.007), respectively，indicating that the patients who administered NE had a higher evacuation rate than those who underwent craniotomy in the RCT and non-RCT group. However, heterogeneity was significant among articles, with *I*^2^ = 92% (*p* < 0.00001) and *I*^2^ = 92% (*p* < 0.00001) in the RCT and non-RCT groups, respectively, and a random-effects model was used (Fig. 5).

#### Duration of operation in the NE and craniotomy groups

**Fig. 6:**
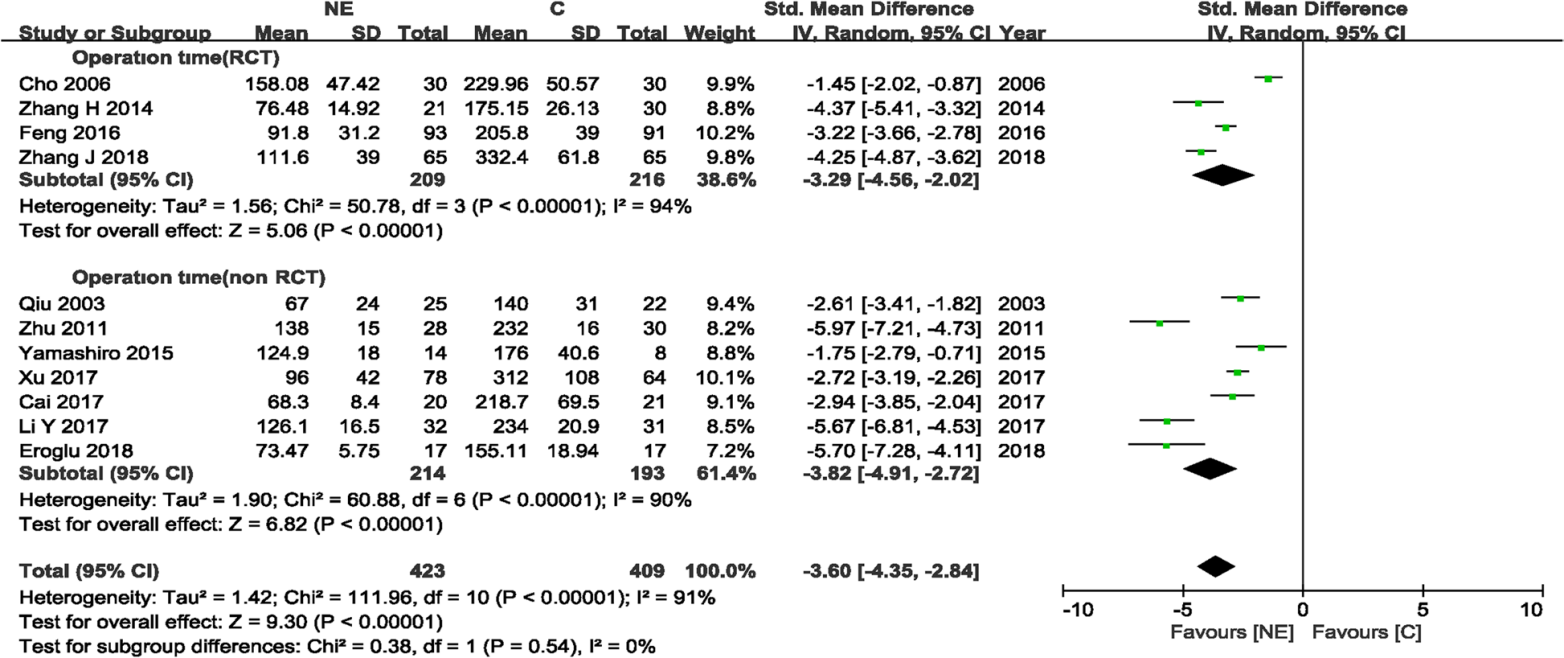
Duration of operation in NE and craniotomy groups

A total of 11 trials [10–13, 15, 16, 19–23] (4 RCTs and 7 non-RCTs) comprising 832 patients (423 and 409 patients in the experimental and control arms, respectively) assessed operation time. There were significant differences in operation time for the RCT and non-RCT groups, with SMD = − 3.29(95%CI − 4.56 to − 2.02, *p* < 0.00001) and SMD = − 3.82 (95%CI − 4.91 to − 2.72, *p* < 0.00001), respectively, indicating that the patients administered NE had reduced operation time compared with the craniotomy group in RCTs and non-RCTs, with *I*^2^ = 94% (*p* < 0.00001) and *I*^2^ = 90% (*p* < 0.00001), respectively; a random-effects model was used for analysis (Fig. 6). Sensitivity analyses also indicated a statistical difference between the two groups, and the funnel plot was visually symmetric (Fig. 3).

#### Good recovery in the NE and craniotomy groups

**Fig. 7:**
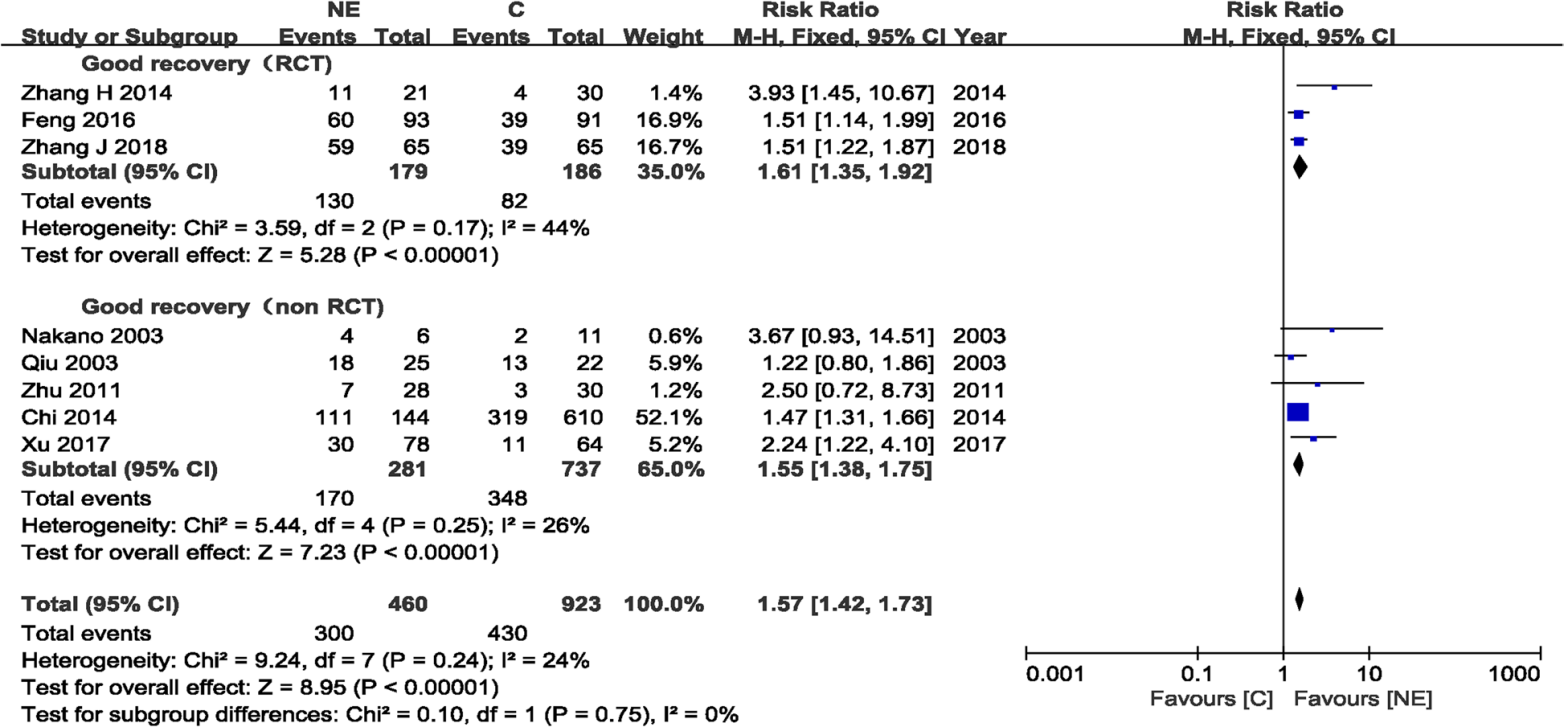
Good recovery in the NE and craniotomy groups

Eight trials [11, 17, 23] assessing 1383 patients (460 and 923 patients in the experimental and control arms, respectively) were included in the meta-analysis of good recovery. There were significant differences in good recovery for the RCT and non-RCTs groups, with RR = 1.61 (95%CI 1.35–1.92, *P* < 0.00001) and RR = 1.55 (95%CI 1.38–1.75, *P* < 0.00001), respectively. Eight studies documented good recovery, and the overall effect showed more patients with good recovery in the NE than C group (RR = 1.57, 95%CI 1.42− 1.73, *p* < 0.00001). There was no evidence of statistically significant heterogeneity (*p* = 0.75 and *I*^2^ = 0%), and a fixed-effects model was used (Fig. 7).

#### Mortality in NE and craniotomy groups

**Fig. 8:**
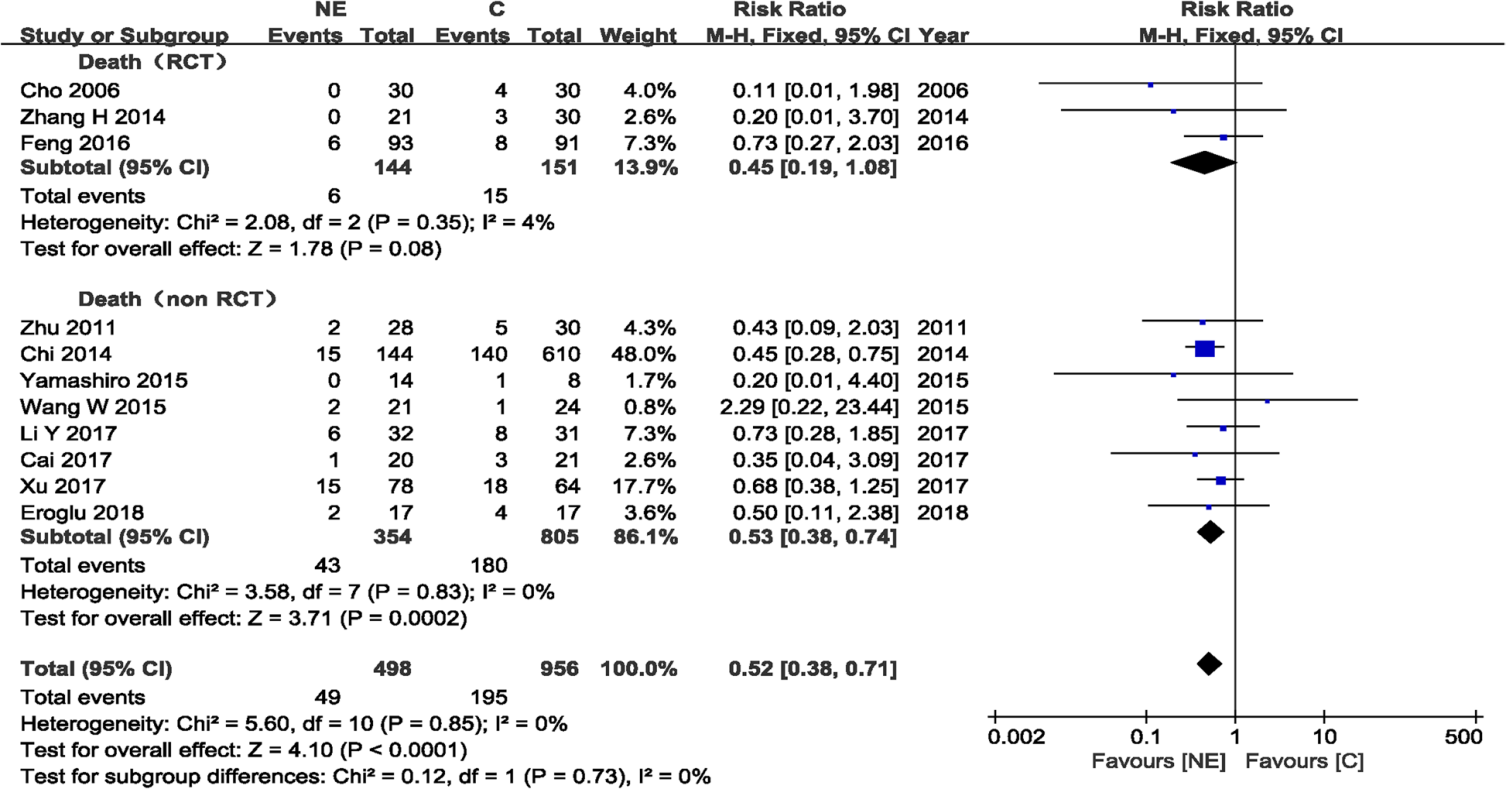
Mortality in NE and craniotomy groups

A total of 11 trials [10–12, 16–23] evaluating 1454 patients (498 and 956 patients in the experimental and control arms, respectively) found mortality rates of 9.8% (49/498) and 20.4% (195/956) in the NE and craniotomy groups, respectively, after NE or craniotomy. The effect of NE or craniotomy on death at the end of follow-up was available in each included study. The pooled RRs of death at the end of follow-up using NE compared to craniotomy for the RCT and the non-RCT groups showed values of 0.45 (95%CI 0.19–1.08, *P* = 0.08) and 0.53 (95% CI 0.38–0.74, *P* = 0.0002), respectively, indicating that patients who underwent NE had a lower mortality rate than craniotomy cases in non-RCTs, while there was no significant difference in the RCTs group. There was no evidence of statistically significant heterogeneity (*p* = 0.73 and *I*^2^ = 0%), and a fixed-effects model was used (Fig. 8). Although the total number of studies in this meta-analysis was small, a symmetrical distribution of funnel plots was observed, suggesting no publication bias (Fig. 2).

#### Additional analyses

Table 3 gives pooled RRs and corresponding 95% CI for the association between neuroendoscopy and craniotomy in ICH patients, according to selected subgroups. There were significant differences between NE group and craniotomy (C) group regarding epilepsy (*p* = 0.01), pneumonia (*p* < 0.00001), hypoproteinemia (*p* = 0.01), tracheotomy (*p* = 0.003), length of ICU stays (p=0.002), length of hospital stays (*p* = 0.02), hospital expenses (*p* < 0.0001), intraoperative blood loss volume (*p* < 0.00001), with NE group having a higher rate of good recovery than craniotomy group (postoperative BI, mRS, and GOS score). In addition, we also evaluated the incidence of intracranial or wound infections (*p* = 0.27), digestive diseases (*p* = 0.72), shunt surgery (*p* = 0.37), postoperative residual hematoma volume (*p* = 0.11) of NE and craniotomy groups, and no significant differences were found between these two groups.

**Table 3:** Pooled RRs and 95% CI for the association between neuroendoscopy and craniotomy in SICH patients, according to selected subgroups

Subgroups	No. of studies	No. of cases	RR (95% CI)	*I*^2^	*P*
Intracranial or wound infection	3	169 (79 cases and 90 controls)	0.45 (95%CI 0.11–1.84)	0%	0.27
Epilepsy	3	281 (142 cases and 139 controls)	0.47 (95%CI 0.26–1.21)	0%	0.01
Pneumonia	7	491 (241 cases and 250 controls)	0.37 (95%CI 0.25–0.56)	0%	< 0.00001
Digestive disease	2	218 (110 cases and 108 controls)	0.79 (95% CI 0.22–2.81)	0	0.72
Hypoproteinemia	1	184 (93 cases and 91 controls)	0.40 (95%CI 0.19–0.82)	Not applicable	0.01
Tracheotomy	2	218 (110 cases and 108 controls)	0.43 (95%CI 0.24–0.76)	0%	0.003
Shunt surgery	2	79 (38 cases and 41 controls)	0.66 (95%CI 0.27–1.62)	0%	0.37
Duration of ICU stay	2	123 (62 cases and 61 controls)	− 0.58 (95%CI − 0.94 to − 0.21)	0%	0.002
Duration of hospital stay	3	205 (110 cases and 95 controls)	− 0.34 (95%CI − 0.61 to − 0.06)	40%	0.02
Hospital costs	2	123 (62 cases and 51 controls)	− 0.75 (95%CI − 1.12 to − 0.38)	0%	< 0.0001
Residual hematoma volume	4	211 (107 cases and 104 controls)	− 0.38 (95%CI − 0.85 to 0.09)	65%	0.11
Blood loss volume	5	553 (285 cases and 268 controls)	− 2.56 (95%CI − 3.44 to − 1.68)	93%	< 0.00001
Postoperative BI score	2	190 (95 cases and 95 controls)	1.48 (95%CI 1.16–1.80)	0%	< 0.00001
Postoperative mRS score	2	193 (99 cases and 94 controls)	− 0.44 (95%CI − 0.73 to − 0.15)	0%	0.003
Postoperative GOS score	2	114 (53 cases and 61 controls)	0.46 (95%CI 0.08–0.83)	0%	0.02

## Discussion

Intracerebral hemorrhage is a common and devastating disease, which requires improved treatment. Surgical treatment of supratentorial intracranial hematoma has the advantages [28] of reducing intracranial pressure, preventing herniation, eliminating the source of hemorrhage, reducing the source of localized mass lesions, and mitigating secondary neuro-inflammatory cascades. Although clinical guidelines [29] for intracranial hemorrhage are widely used, accompanying factors [30, 31] such as patient age, the Glasgow Coma Scale (GCS) score at admission, and hematoma volume, depth and location, usually influence the neurosurgeon’s decision regarding surgical treatment. Currently, there are many surgical procedures for treating intracranial hematoma, including traditional craniotomy, stereotactic aspiration, and endoscopic surgery. Compared with craniotomy, endoscopic surgery has direct vision and less damage to the surrounding normal brain tissue, and is highly recommended by many neurosurgeons [32]. However, indiscriminate restriction of the ICH indication based solely on dominance criteria could reduce the odds of patient survival in some cases.

The main complications of ICH are re-bleeding, intracranial infection, and pulmonary infection. In this study, the observation group’s incidence of postoperative complications was noticeably lower than that of the control group. The main reasons were analyzed as follows: (1) the traditional craniotomy is more traumatic and causes irreversible damage to the brain tissue and blood vessels in a fistula. Neuroendoscopic surgery is more consistent with the concept of minimally invasive surgery and can effectively avoid important brain functional areas. (2) Craniotomy adopts exterior lighting that is not bright enough for deep hematoma, while neuroendoscopy uses internal lighting, which allows for close observation. The brightness remains unchanged even with the changes in the hematoma depth, which improves the procedure’s accuracy by clearly displaying the intraoperative condition. (3) The infection risk is reduced due to the short operation time, small incision, and minor brain tissue damage.

A systematic review published in 2017 noted [27] that patients with ICH may benefit more from endoscopic surgery than from craniotomy, which supports the current study. In comparison to craniotomy, neuroendoscopic surgery has the advantages of higher hematoma evacuation rate, shorter operation time, better prognosis, and lower mortality.

However, some data included in this analysis were incorrect, such as data regarding patients with rebleeding (NE vs. C, 1 vs. 3) in Cho’s study [10] and death in Feng’s study [12] (NE vs. C, 6 vs. 8), although this did not affect the overall results. Furthermore, this study provided a different point of view in terms of re-bleeding and hospital stay duration; in addition, we also assessed the incidence of shunt surgery and the improvement of postoperative BI, GOS and GCS score, hospital costs, etc., for patients who administered neuroendoscopic surgery or craniotomy, adding new findings into this study. Endoscopic craniotomy with small bone window does not require the use of artificial materials (such as artificial dura mater) that are necessary for an operation and is not subject to secondary cranioplasty, hospitalization therefore cost less. In conclusion, this may serve as a constructive guideline for neurosurgeons in selecting the surgical procedure for treating intracranial hemorrhage. We found that endoscopic surgery, as opposed to craniotomy, can improve patient prognosis.

In this review, statistical heterogeneity was found between endoscopic surgery and craniotomy, in terms of operation time, hematoma residual volume, intraoperative blood loss, and hematoma evacuation, so a random-effects model and the jack-knife method were used to analyze pooled data with high heterogeneity.

In this review, statistical heterogeneity was found between endoscopic surgery and craniotomy in terms of operation time, hematoma residual volume, intraoperative blood loss, and hematoma evacuation, so a random-effect model and the jack-knife method were used to analyze pooled data with high heterogeneity.

For operative time analysis, when the studies by Cho and Feng [10, 12] in RCTs were excluded, further analysis showed that there was no heterogeneity (*P* = 0.85; *I*^2^ = 0%). But heterogeneity remained in the non-RCT group and the overall group; similar results were obtained for the overall effect (data not shown). When compared to the traditional craniotomy, neuroendoscopic surgery has the advantages of small incision and simple operation with the endoscopic working channel, thus reducing the operation time.

Neuroendoscopic minimally invasive surgery with small incision and small opening of bone window can effectively reduce traumatic injuries to patients. A clear surgical field helps prevent damage to normal tissues surrounding a lesion, shorten the operation time, avoid brain tissue being massively exposed for a long period of time, reduce stress reactions, and lower the risk of cerebral edema. The hematoma can be precisely located and effectively removed using neuroendoscopic observation in conjunction with CT positioning, and the removal process is regulated and safer with constant speed, which is beneficial to lower the risk of reperfusion injury and protect cerebral vessels and cranial nerve tissue.

The more the residual hematoma during operation, the worse the operative outcome. We found that there was no statistical heterogeneity for hematoma residual volume in non-RCTs (*p* = 0.61; *I*^2^ = 0%) after excluding Li Y’s study [21] and different results were observed for the overall effect. NE had a higher evacuation rate compared with the craniotomy groups, with SMD = − 0.59 (95%CI − 0.92 to − 0.26, *p* = 0.0005) (data not shown).

Theoretically, massive intraoperative blood loss may lead to hypoproteinemia or anemia after surgery. We found that the rate of hypoproteinemia after NE was lower than upon craniotomy，with no statistical heterogeneity for intraoperative blood loss volume in non-RCTs (*p* = 0.65; *I*^2^ = 0%) after excluding Xu’s study [23]. However, heterogeneity remained in the overall population; similar results were found for the overall effect, with SMD = − 3.00 (95%CI − 4.20 to − 1.80, *p* < 0.00001) (data not shown).

Enlargement of intracerebral hemorrhage is the main cause of early clinical deterioration. About 20–40% of the patients show hematoma re-expansion within the first 24h after hemorrhage [33]. Large amounts of hematoma are one of the causes of poor prognosis and high mortality. Previous findings indicate that NE has a high evacuation rate, from 79.2 to 99%, with significant difference compared with craniotomy [12, 26, 34, 35]. Theoretically, surgical hematoma evacuation would benefit patients. We used the jack-knife method to perform sensitivity analysis in the hematoma evacuation group. Therefore, the meta-analysis was repeated four and five times, respectively, each omitting a different study; finally, there was no statistical significance after excluding Zhang J’s article [13] in the RCT group and Zhu or Eroglu’s article [16, 22] in the non-RCT group. However，the same results were obtained for the overall effect (data not shown).

A possible reason for heterogeneity is that this study included multicenter trials, with differences in surgical procedures and treatments in many countries or different hospitals in the same country potentially leading to heterogeneity. We performed two subgroup analyses according to country and publication year, for preoperative Glasgow Coma Scale score and hematoma volume, and similar results were obtained in this work (data not shown).

In this study，the total rebleeding rate was significantly lower in the endoscopy group (3.5%; 8/227) compared with the craniotomy group (9.3%; 21/226), in disagreement with a previous publication [27] (2017). In addition, we assessed complications, including the rates of rebleeding, wound and intracranial infection, epilepsy, pneumonia, digestive tract disease, tracheotomy, hypoproteinemia, and shunt surgery respectively, as well as the incidence of total complications. There was no significant heterogeneity among articles, with *I*^2^ = 0% (*p* = 1.0) in total complications. In the NE group, 8.0% (84/1051) of patients had complications, while 18.7% (199/1066) was found in the craniotomy group. Pooled analysis showed that occurrence of total complications between the NE and craniotomy groups showed a significant difference (*p* < 0.00001, data not shown). The higher complications in the craniotomy group may be due to longer operation time, larger damage and elevated blood loss.

The cost for treating ICH was reported to be high, up to more than $44,000 in the first year of treatment alone [36]. In the current trials, NE incurred less hospital expenses compared with craniotomy due to shorter hospital stay, lower rate of complications, shorter operation time and better recovery in the latter procedure.

Zhang [13] mentioned that SP (serum substance P) and IL-2 levels in the NE group are significantly higher than control values four weeks after the operation, while IL-6, hs-CRP (high sensitive C-reactive protein), TNF-α (tumor necrosis factor-α) and SF (serum ferritin) levels are significantly lower compared with the craniotomy group. These results showed that endoscopic surgery effectively promotes the recovery of damaged glial cells and is helpful for the prognostic rehabilitation of patients.

The findings show that minimally invasive neuroendoscopic surgery can effectively lower the risk of complications, promote the recovery of neurological function, and improve patients’ life quality. This may be attributed to the minor harm that minimally invasive neuroendoscopic surgery causes to brain tissue. Brain tissue can avoid being massively exposed for a long period of time due to small incision, tiny bone foramen, and short operation time, thus reducing the chance of intracranial and pulmonary infections, intracranial re-bleeding, upper gastrointestinal hemorrhage, and other complications, effectively relieving brain tissue damage caused by cerebral hemorrhage and cerebral edema as well as decreasing the risk of death. Moreover, minimally invasive surgery can effectively reduce the stress stimulation of surgical operation on a body, lessen the pathological damage to brain tissue, relieve the pain of patients, shorten the ICU stay length and speed up the recovery of the patient's neurological function, thus improving the patient’s life quality [37–39].

In addition, different surgical approaches may improve the outcome of patients with ICH [40]. Based on previous reports and our own experience, we believe that a single surgical procedure cannot be fully adapted to all patients, and the procedure should be selected dialectically. Endoscopic surgery combined with stereotactic navigation, 3D reconstruction, intraoperative CT imaging, B-ultrasound or other techniques may cause more patients to benefit from this operation [41–43].

Limitations of this meta-analysis must be pointed out. Firstly, some of the included trials were non-RCTs, and most studies did not report random sequence generation and allocation concealment. Secondly, the duration of follow-up differed in these studies. Therefore, more studies addressing complications, good recovery, and mortality with uniform follow-up times of at least 6 months are required. Thirdly, the number of included patients was relatively limited in this review, which may affect the obtained results. Furthermore, heterogeneity was found in the pooled data for operation time，evacuation rate, residual hematoma volume and intraoperative blood loss volume, and a random-effects model was used to estimate the overall effects more conservatively.

## Conclusion

Neuroendoscopic surgery is associated with significantly reduced complication and death rates after surgical evacuation of ICH. There was also a statistically significant reduction in the risk of poor functional outcome after neuroendoscopy. These findings clearly demonstrate the advantages of neuroendoscopic surgery for ICH treatment. This study could guide clinicians in selecting treatment options and appropriate patients for neuroendoscopic surgery in ICH. However, further randomized controlled trials are required to control all confounding factors and confirm this conclusion. Meanwhile, neurosurgeons should also improve their surgical skills to reduce the impact of human factors in surgical procedures.

## Data Availability

The datasets used and/or analyzed during the current study are available from the corresponding author on reasonable request.

## Abbreviations

ADL: Activities of daily living
BI: Barthel index
C: Craniotomy
CI: Confidence interval
GCS: Glasgow Coma Scale
GFO: Good functional outcome
GOS: Glasgow Outcome Scale
RCT: Randomized controlled trial
RR: Relative risk ratio
mRS: Modified Rankin Scale
NE: Neuroendoscopy
SICH: Spontaneous intracerebral hemorrhage
SMD: Standard mean difference
